# Usp7-dependent histone H3 deubiquitylation regulates maintenance of DNA methylation

**DOI:** 10.1038/s41598-017-00136-5

**Published:** 2017-03-03

**Authors:** Luna Yamaguchi, Atsuya Nishiyama, Toshinori Misaki, Yoshikazu Johmura, Jun Ueda, Kyohei Arita, Koji Nagao, Chikashi Obuse, Makoto Nakanishi

**Affiliations:** 1 Department of Cell Biology, Graduate School of Medical Sciences, Nagoya City University, 1, Kawasumi, Mizuho-cho, Mizuho-ku, Nagoya 467-8601 Japan; 2 Division of Cancer Cell biology, Department of Cancer Biology, Institute of Medical Science, University of Tokyo, Tokyo, 108-8639 Japan; 3 Center for Education in Laboratory Animal Research, Chubu University, Kasugai, Aichi 487-8501 Japan; 4 Graduate School of Medical Life Sciences, Yokohama City University, 1-7-29, Suehiro-cho, Tsurumi-ku, Yokohama 230-0045 Japan; 5 Division of Molecular Life Science, Graduate School of Life Science, Hokkaido University, Sapporo, 001-0020 Japan

## Abstract

Uhrf1-dependent histone H3 ubiquitylation plays a crucial role in the maintenance of DNA methylation via the recruitment of the DNA methyltransferase Dnmt1 to DNA methylation sites. However, the involvement of deubiquitylating enzymes (DUBs) targeting ubiquitylated histone H3 in the maintenance of DNA methylation is largely unknown. With the use of *Xenopus* egg extracts, we demonstrate here that Usp7, a ubiquitin carboxyl-terminal hydrolase, forms a stable complex with Dnmt1 and is recruited to DNA methylation sites during DNA replication. Usp7 deubiquitylates ubiquitylated histone H3 *in vitro*. Inhibition of Usp7 activity or its depletion in egg extracts results in enhanced and extended binding of Dnmt1 to chromatin, suppressing DNA methylation. Depletion of Usp7 in HeLa cells causes enhanced histone H3 ubiquitylation and enlargement of Dnmt1 nuclear foci during DNA replication. Our results thus suggest that Usp7 is a key factor that regulates maintenance of DNA methylation.

## Introduction

In higher eukaryotes, DNA methylation plays a crucial role in many biological processes, such as transcriptional regulation of developmental genes, silencing of transposable elements, and X-chromosome inactivation, and is involved in maintenance of genomic integrity and tumorigenesis^1–4^. In mammals, DNA methyltransferases 3A and 3B (Dnmt3A and Dnmt3B) catalyze *de novo* DNA methylation of unmethylated DNA. Once a DNA methylation pattern is established, it is stably maintained during cell proliferation by so-called maintenance DNA methylation, mainly catalyzed by Dnmt1^5, 6^. During DNA replication, Uhrf1, a Dnmt1 interacting protein, is recruited to chromatin via its hemi-methyl DNA binding activity, and promotes the loading of Dnmt1 onto chromatin^7–11^. Then, Dnmt1 converts hemi-methylated DNA to fully-methylated DNA in a processive manner^12, 13^. This conversion finally displaces both Uhrf1 and Dnmt1 from chromatin.

The use of *Xenopus* egg extracts is a powerful *in vitro* cell-free system for the analysis of chromosomal replication, in which replication complexes successfully assemble on exogenously-added sperm chromatin, leading to a single round of chromosomal replication in the absence of transcription^14, 15^. We previously reported that interphase *Xenopus* egg extracts successfully recapitulate Uhrf1-dependent DNA methylation *in vitro,* and Uhrf1-dependent histone H3 ubiquitylation is necessary for Dnmt1 recruitment to sites of DNA methylation^16^. Ubiquitylated histone H3 directly interacts with the RFTS domain of Dnmt1, possibly through its UIM motif^17^. Interestingly, ubiquitylated histone H3 disappears when DNA replication is completed. Conversely, this ubiquitylation greatly accumulates when Dnmt1 function is aberrant^16^. These results suggest that histone H3 ubiquitylation might regulate the dynamic action of Dnmt1 during the maintenance of DNA methylation.

Protein deubiquitylation is a complicated, highly-regulated process modulated by numerous deubiquitylating enzymes (DUBs)^18, 19^. Although much progress has been made in determining how histone ubiquitylation is achieved and regulated, the importance of the reversal of this modification has only recently been appreciated. So far among over 100 DUBs, multiple DUBs have been shown to target ubiquitylated histones H2A and H2B^20, 21^. However, it is currently not known which DUBs target histone H3. One such candidate is Ubiquitin-specific protease 7 (USP7), also known as HAUSP. In mammalian cells, Usp7 forms a complex with Dnmt1 and/or Uhrf1, and regulates the stability of these proteins by competing with the Uhrf1-dependent polyubiquitylation and subsequent proteasomal degradation of these proteins^22, 23^. Therefore, Usp7 promotes DNA methylation by increasing the amount of Dnmt1^24^. However, the mode of action of Usp7 in the maintenance of DNA methylation remains elusive.

In this study, we show that Usp7 is involved in the maintenance of DNA methylation in both *Xenopus* and mammals. Treatment with a specific Usp7 inhibitor or Usp7 depletion results in reduction of DNA methylation efficiency. Our results thus suggest that histone H3 deubiquitylation by Usp7 is an important step in the maintenance of DNA methylation.

## Results

### Inhibition of pan-DUB activity blocks efficient DNA methylation in *Xenopus* egg extracts

**Figure 1:**
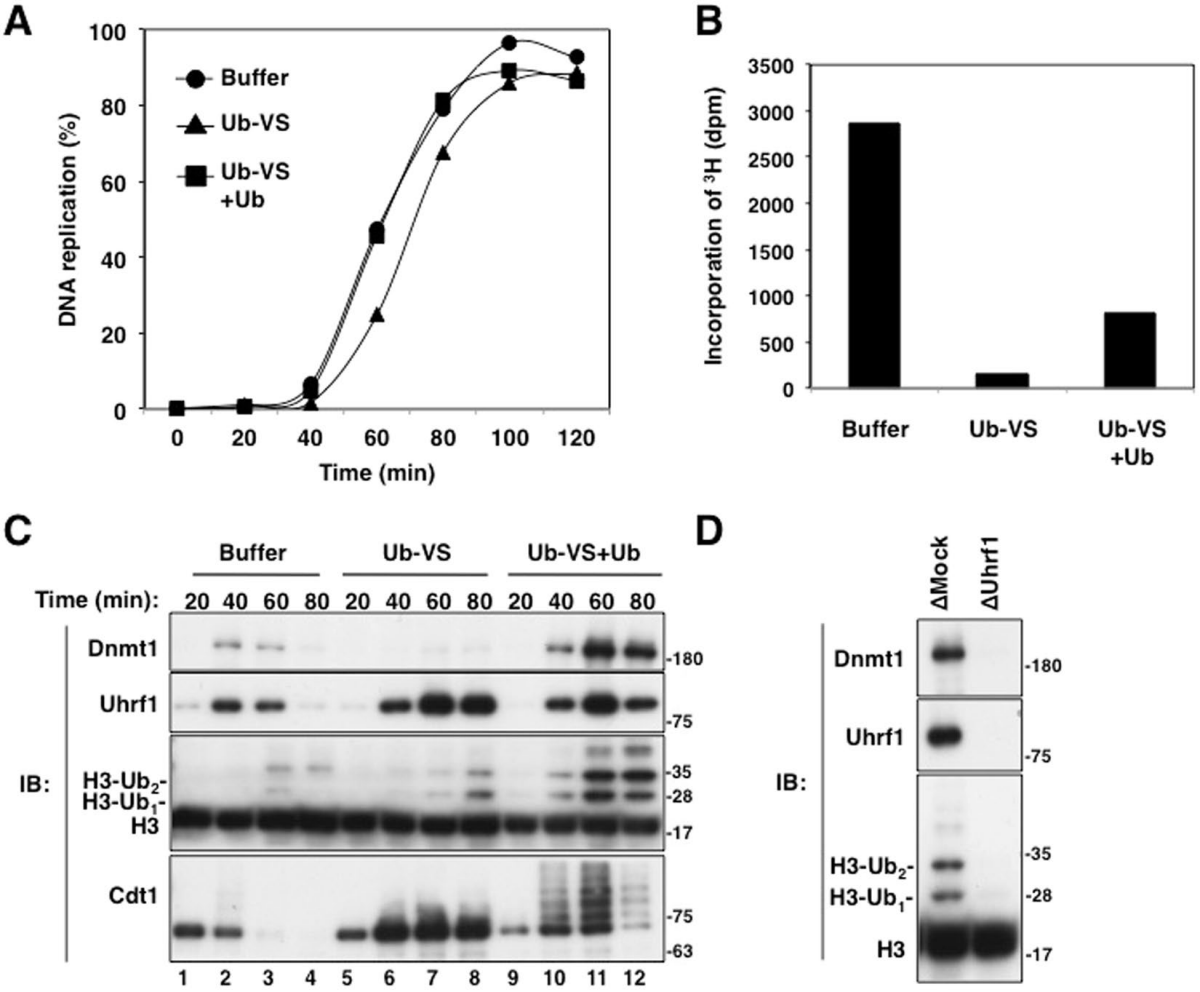
DUBs activity is important for maintenance of DNA methylation, but not for DNA replication, in *Xenopus* egg extracts. (**A**,**B**) Sperm chromatin was incubated with interphase *Xenopus* egg extracts supplemented with buffer (+buffer), 20 μM Ub-VS (+Ub-VS), or 20 μM Ub-VS and 0.2 mg/ml ubiquitin (+Ub-VS +Ub). Prior to addition of sperm chromatin, radiolabeled [α-^32^P]dCTP (**A**) or S-[methyl-^3^H]-adenosyl-L-methionine (**B**) was added to extracts. Purified DNA samples were analyzed to determine the efficiency of DNA replication (**A**) and DNA methylation at 90 min after addition of sperm DNA (**B**), as described in Experimental Procedures. (**C**) Interphase egg extracts were treated as in (**A**) and chromatin fractions were isolated and subjected to immunoblotting using the antibodies indicated. (**D**) Mock- or Uhrf1-depleted interphase egg extracts were treated with Ub-VS and ubiquitin as in (**A**). ∆ Denotes immunodepletion of the protein indicated. The resultant chromatin fractions were subjected to immunoblotting using the antibodies indicated. Source data are available online for this figure.

Although histone H3 ubiquitylation is critical for recruitment of Dnmt1 to DNA replication sites, it is hardly detectable during normal S-phase. Accumulation of this ubiquitylation in the absence of Dnmt1 (Supplementary Figure S1) suggests that ubiquitylated histone H3 is rapidly turned over after conversion of hemi-methylated DNA to fully-methylated DNA. To examine whether certain DUB(s) are involved in this rapid process, we first examined the effect of ubiquitin-vinyl-sulfone (Ub-VS), a pan-inhibitor of only active DUBs^25^, on histone H3 ubiquitylation and maintenance DNA methylation. The maintenance of DNA methylation was evaluated by assessing the incorporation of [^3^H]-S-Adenosyl-L-methionine into sperm DNA. In our previous work, we demonstrated that the incorporation of radioactivity in sperm DNA was dependent on ongoing DNA replication, indicating that our assay system specifically measured maintenance DNA methylation but not *de novo* DNA methylation^16^. As reported previously, in *Xenopus* egg extracts inhibition of active DUBs by Ub-VS resulted in suppression of global protein ubiquitylation, due to the inhibition of free ubiquitin recycling and the subsequent starvation of free ubiquitin (see Fig. 1C). Addition of free ubiquitin to Ub-VS-treated extracts specifically restores ubiquitylation activity, while all deubiquitylation remains inhibited^26, 27^. Although treatment of egg extracts with Ub-VS did not affect DNA replication, as reported previously^27^ (Fig. 1A), it significantly inhibited DNA methylation (Fig. 1B). These observations were also observed in two additional independent experiments (Supplementary Fig. S2A,B). It should be noted that the treatment of egg extract with Ub-VS or depletion of proteins from the egg extract as well as mock depletion delayed DNA replication timing, presumably due to dilution of interphase egg extract by the drug treatment or immunodepletion procedures, which was usually observed in this *in vitro* cell-free system^30^. However, it did not significantly affect the efficiency of DNA replication and chromatin loading of xPCNA, indicating that treatment with Ub-VS did not affect DNA replication. Furthermore, we found that addition of free ubiquitin to Ub-VS-treated extracts did not effectively restore DNA methylation (Fig. 1B). These results support our previous finding that histone H3 ubiquitylation is critical for DNA methylation maintenance^16^, and also suggest that a DUB-dependent process is important for efficient DNA methylation.

When egg extracts were treated with Ub-VS, DNA replication-dependent Cdt1 ubiquitylation and its degradation were completely abolished (Fig. 1C, compare lanes 1–4 and 5–8), indicating that all protein ubiquitylation was indeed inactivated, presumably due to starvation of free ubiquitin. In Ub-VS-treated extracts, chromatin binding of Dnmt1 and dissociation of Uhrf1 were significantly suppressed. These results are consistent with impaired DNA methylation in Ub-VS-treated extracts (Fig. 1C, lanes 5–8, see also Fig. 1B). When an excess amount of free ubiquitin was added to Ub-VS-treated extracts, the ubiquitylation of Cdt1 as well as histone H3 were greatly restored (Fig. 1C, lanes 9–12). This ubiquitylation of histone H3 was completely dependent on the presence of Uhrf1 (Fig. 1D). In contrast, addition of ubiquitin still failed to dissociate Uhrf1 from chromatin during this experimental period (Fig. 1C, compare lanes 1–4 and 9–12). Again, these results suggest that DUB activity is required for maintenance of DNA methylation. Consistent with this, specific inhibition of DUB activity resulted in hyper-accumulation of Dnmt1 on chromatin (Fig. 1C, compare lanes 5–8 and 9–12). Accumulation of Dnmt1 on chromatin in Ub-VS-treated extracts was also dependent on the presence of Uhrf1. Similarly, when egg extracts were treated with PR-619, a pan-DUB inhibitor, dissociation of both Dnmt1 and Uhrf1 from chromatin was markedly compromised, and DNA methylation was suppressed (Supplementary Fig. S3A,B). Notably, inhibition of proteasome activity by MG132 treatment did not greatly affect the kinetics of chromatin binding of Dnmt1 and Uhrf1 (Fig. S3C), although it slightly increased the amounts of Dnmt1 and Uhrf1 on chromatin, and inhibited Cdt1 degradation, as reported previously^28^. Given that treatment with Ub-VS almost completely abolished DNA methylation, the results suggest that the effect of Ub-VS on DNA methylation is independent of proteasomal degradation.

### Usp7 interacts with Dnmt1 in *Xenopus* egg extracts

**Figure 2:**
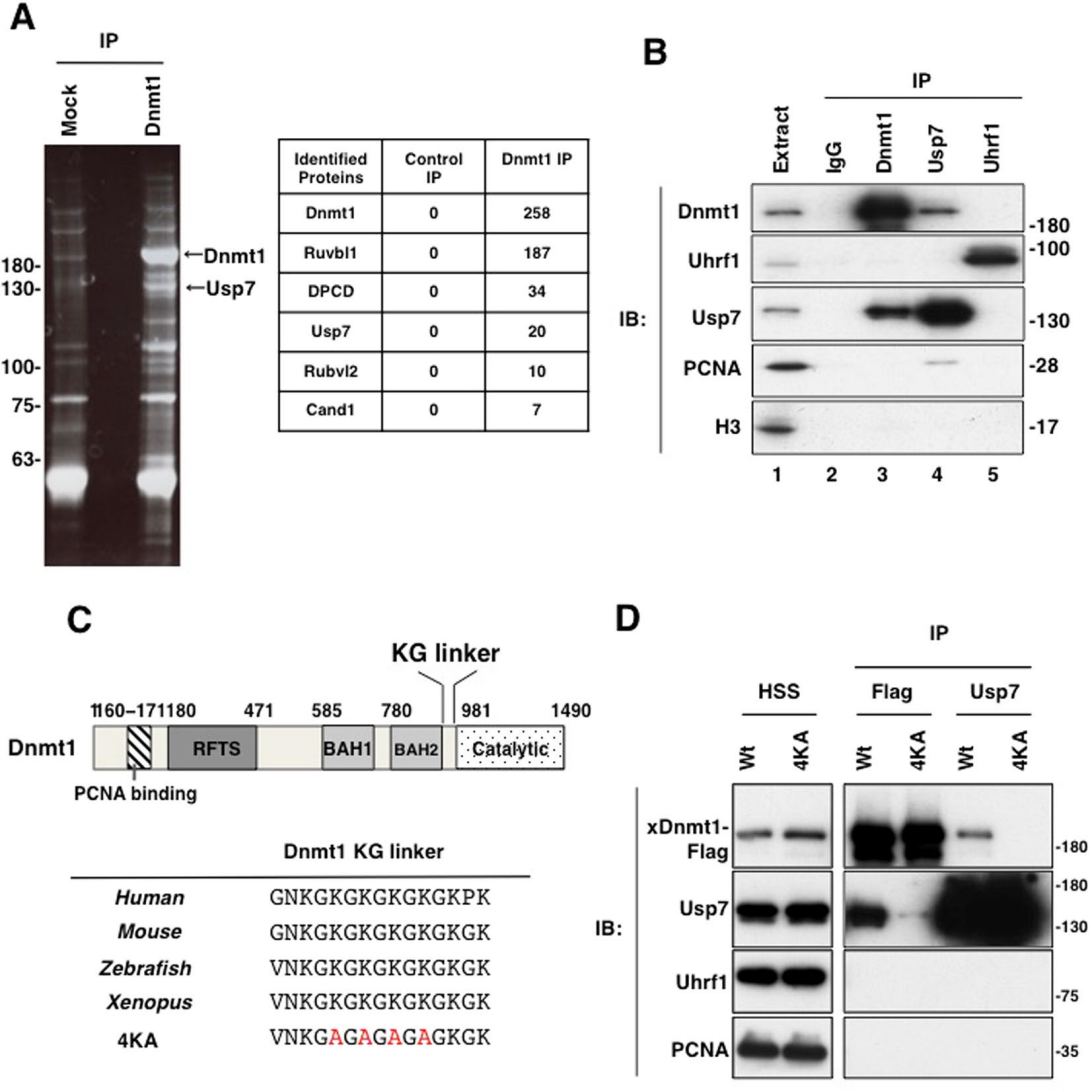
Usp7 forms a stable complex with Dnmt1 in *Xenopus* egg extracts. (**A**) Dnmt1 was immunoprecipitated from interphase HSS using specific antibodies. The associated polypeptides were identified by mass spectrometry. The resultant immunoprecipitates were subjected to SDS-PAGE and stained with a SyproRuby; the peptides corresponding to Dnmt1 and Usp7 are indicated. The numbers of unique spectra identified for each protein are shown in the table. (**B**) Immunoprecipitates using anti-Dnmt1 (lane 3), anti-Usp7 (lane 4), anti-Uhrf1 (lane 5) antibodies, or control IgG (lane 2) as well as egg extracts (lane 1) were subjected to immunoblotting using the antibodies indicated. (**C**) Sequence alignment of the KG linker of Dnmt1 across different species. Red denotes residues mutated in a 4KA mutant used in this study. (**D**) FLAG-tagged wild-type or a 4KA mutant of recombinant *Xenopus* Dnmt1 was introduced into *Xenopus* egg extracts. Recombinant or endogenous Usp7 was immunoprecipitated using either anti-FLAG-M2 agarose beads or protein A agarose beads conjugated with anti-Usp7 antibodies, respectively. Immunoprecipitates were then subjected to immunoblotting using the antibodies indicated. Source data are available online for this figure.

Given that Dnmt1 depletion resulted in accumulation of ubiquitylated histone H3 (Supplementary Fig. S1), we speculated that certain DUB(s) might form a stable complex with Dnmt1. We thus performed mass spectrometry analysis using immunopurified Dnmt1 complex from egg extracts. This analysis identified several proteins including Usp7 as binding partners of Dnmt1 (Fig. 2A). The specificity of the interaction between Dnmt1 and Usp7 was validated by a reciprocal immunoprecipitation experiment using antibodies specific for these two proteins (Fig. 2B). Furthermore, purified FLAG-tagged recombinant *Xenopus* Dnmt1 produced in insect cells specifically bound to endogenous Usp7 in interphase egg extracts. This interaction was compromised by amino acid substitutions of Dnmt1 in the KG linker (xDnmt1 4KA), although this mutant retained a trace ability to bind to Dnmt1. This region was recently identified as a highly-conserved Usp7-binding region^23^ (Fig. 2C,D). In contrast, we failed to detect Uhrf1 in Dnmt1 and Usp7 complexes (Fig. 2B, lane 5 and 2D). Collectively, these results indicate that Usp7 forms a complex with Dnmt1 in egg extracts.

### Usp7 is recruited to chromatin during DNA replication in a Uhrf1- and Dnmt1-dependent manner

**Figure 3:**
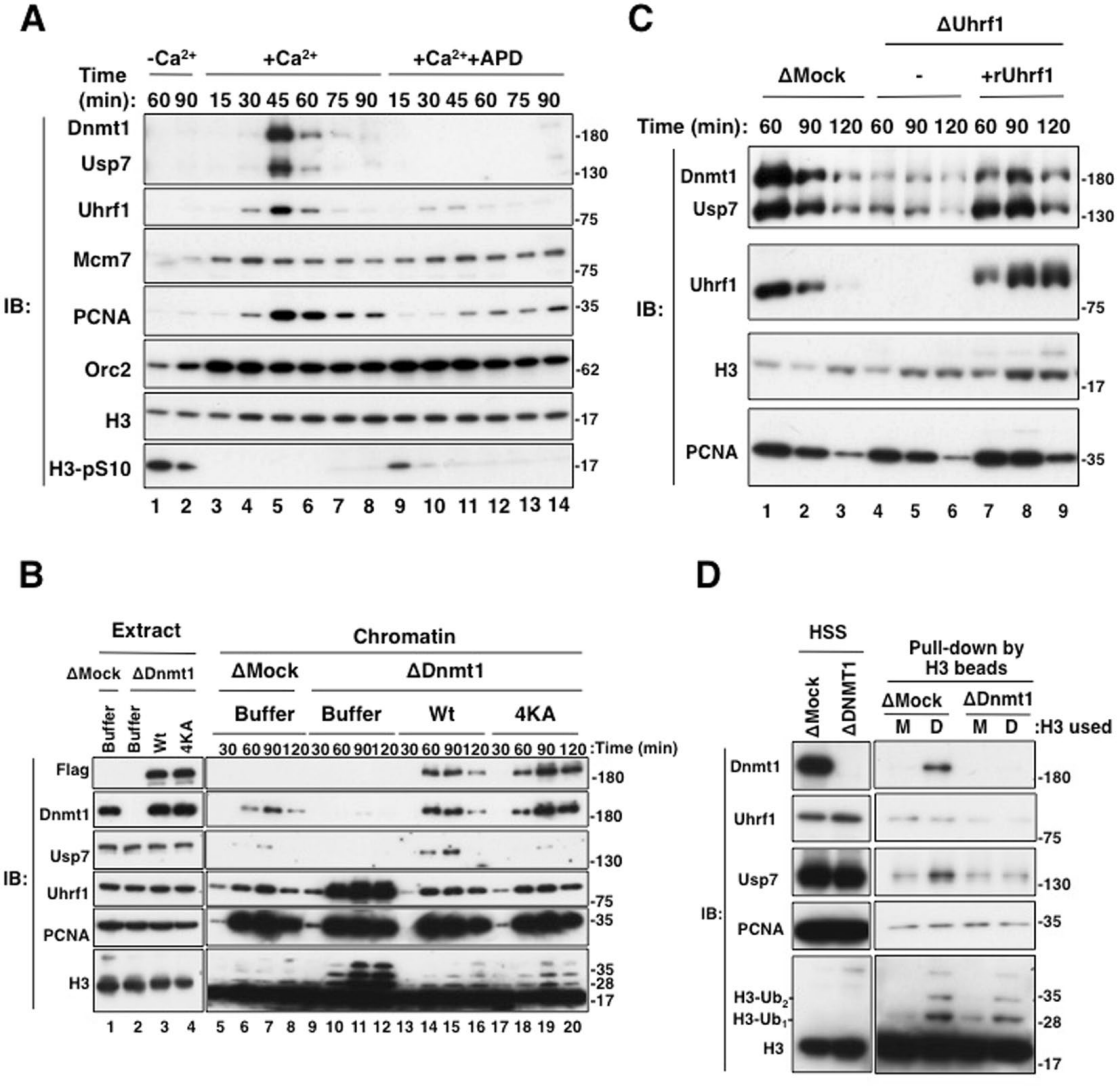
Usp7 binds to replicating chromatin in a Dnmt1-dependent manner. (**A**) CSF extracts (M-phase) were released into interphase by addition of CaCl_2_. Sperm nuclei were added to either CSF or interphase extracts. Aphidicolin (APD, 150 μM) was added to extracts 10 min before sperm addition. At the indicated times after incubation, chromatin fractions were isolated and subjected to immunoblotting using the antibodies indicated. (**B**) Sperm chromatin was added to mock- or xDnmt1-depleted interphase extracts. xDnmt1-depleted extracts were supplemented with either buffer alone (lanes 9–12), purified wild-type xDnmt1-FLAGx3 or its 4KA mutant-FLAGx3 (10 ng/μl final concentration, lanes 13–16 or 17–20, respectively). At the indicated time points, chromatin fractions as well as extracts were subjected to immunoblotting using the antibodies indicated. ∆ Denotes immunodepletion of the protein indicated. (**C**) Sperm chromatin was added to mock-depleted or xUhrf1-depleted interphase extracts. xUhrf1-depleted extracts were supplemented with either buffer alone (lanes 7–9) or purified hUhrf1 (30 ng/μl final concentration, lanes 10–12). At the indicated time points, chromatin fractions were isolated and subjected to immunoblotting using the antibodies indicated. Note that xUhrf1 antibodies recognized hUhrf1. ∆ Denotes immunodepletion of the protein indicated. (**D**) Either mock- or Dnmt1-depleted HSS were incubated with chromatin fractions from mock- (M) or Dnmt1- (**D**) depleted extracts. The resultant mixtures were immunoprecipitated with anti-histone H3 antibodies and subjected to immunoblotting using the antibodies indicated. ∆ Denotes immunodepletion of the protein indicated. Source data are available online for this figure.

We then asked whether Usp7 binds to chromatin in a cell cycle phase-dependent manner. *Xenopus* sperm chromatin was added to cytostatic factor (CSF)-arrested extracts, and then CaCl_2_ was added to trigger the M-phase to interphase transition. Usp7 bound to chromatin specifically from interphase egg extracts. Mcm7, a component of the MCM2-7 complex, bound to chromatin prior to Usp7, while Uhrf1 and Dnmt1 as well as PCNA bound to chromatin with the same kinetics as did Usp7 (Fig. 3A, lanes 3–8). We then examined whether chromatin loading of Usp7 is dependent on DNA replication. The loading of Usp7 onto chromatin was completely suppressed in egg extract when DNA replication was blocked by treatment with geminin, p27^Kip1^ (Supplementary Fig. S4) or aphidicolin (APD) (Fig. 3A, lanes 9–14). Taken together, we conclude that chromatin binding of Usp7 requires ongoing DNA replication.

Given that Usp7 forms a stable complex with Dnmt1, we speculated that chromatin loading of Usp7 is dependent on the presence of Dnmt1. We found that depletion of Dnmt1 markedly compromised chromatin loading of Usp7 (Fig. 3B, compare lanes 5–8 and 9–12). As expected, Dnmt1 depletion resulted in the accumulation of Uhrf1 and histone H3 ubiquitylation on chromatin (Fig. 3B, lanes 9–12). Reintroduction of wild-type xDnmt1 rescued the reduced chromatin loading of Usp7 (Fig. 3B, lanes 13–16). Importantly, reintroduction of the 4KA mutant failed to restore effective chromatin loading of Usp7 (Fig. 3B, lanes 17–20). These results suggest that chromatin loading of Usp7 is dependent on its complex formation with Dnmt1.

As reported previously, the binding of Dnmt1 to chromatin is dependent on the presence of Uhrf1. Therefore, recruitment of Usp7 to chromatin required Uhrf1 as well (Fig. 3C, lanes 4–6). Reintroduction of recombinant hUhrf1 rescued the reduced recruitment of Usp7 on chromatin (Fig. 3C, lanes 7–9).

We then examined whether the Dnmt1-Usp7 complex could bind to ubiquitylated histone H3, because Dnmt1 recruitment to DNA replication sites is dependent on this ubiquitylation. Unmodified or ubiquitylated histone H3 were immunoprecipitated using specific antibodies to histone H3 from MNase-digested mock- or Dnmt1-depleted chromatin samples. The resultant immunoprecipitates were then incubated with high-speed supernatants (HSS) of egg extracts. Dnmt1-Usp7 complex bound to ubiquitylated histone H3 more efficiently when compared with unmodified histone H3 (Fig. 3D, lanes 1 and 2). As expected, Usp7 failed to bind ubiquitylated histone H3 when Dnmt1 was depleted from HSS (Fig. 3D, lanes 2 and 4). These results strongly suggest that Dnmt1-Usp7 is recruited to DNA replication sites via Dnmt1 binding to ubiquitylated histone H3.

### Usp7 depletion increases the level of ubiquitylated histone H3

**Figure 4:**
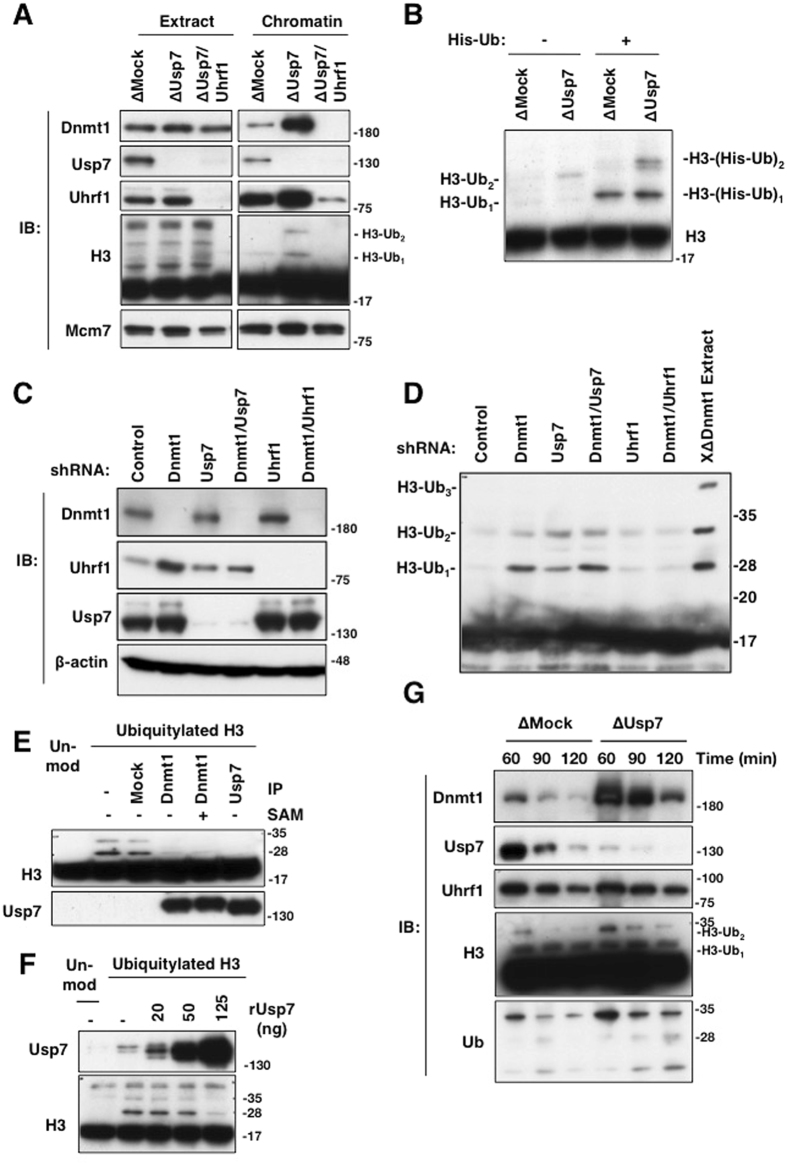
Usp7 depletion and inhibition result in accumulation of ubiquitylated histone H3 and enhancement of Dnmt1 chromatin loading. (**A**) Sperm chromatin was added to either mock-, Usp7-, or Usp7/Uhrf1-double depleted extracts for 90 min. Isolated chromatin fractions as well as extracts were subjected to immunoblotting using the antibodies indicated. ∆ Denotes immunodepletion of the protein indicated. (**B**) Sperm chromatin was added to either mock- or Usp7-depleted extracts for 150 min in the presence or absence of 0.1 mg/ml His_6_-ubiquitin. Chromatin fractions were isolated and subjected to immunoblotting using anti-histone H3 antibodies. ∆ Denotes immunodepletion of the protein indicated. (**C,D**) HeLa cells were infected with lentiviruses expressing tet-on shRNAs targeting Luciferase (Control), Dnmt1, Usp7, and Uhrf1, in the combinations indicated. Cells were then harvested after treatment with doxycycline (1 μg/ml) for 3 days (**C**) and whole cell extracts were subjected to immunoblotting using the antibodies indicated. Histones were acid-extracted from cells depleted of the indicated proteins and subjected to immunobloting using anti-histone H3 antibodies (**D**). xDnmt1-depleted chromatin was used as a positive control. (**E**) USP7 deubiquitylates histone H3 *in vitro*. Dnmt1/Usp7 complex deubiquitylates histone H3 either in the absence or presence of SAM. The indicated immunoprecipitates (IP) were incubated with MNase-digested chromatin containing unmodified (lane1) or ubiquitylated histone H3 in the presence or absence of S-adenosylmetionine (SAM). (**F**) Purified recombinant Usp7 was incubated with ubiquitylated histone H3 for 2 hrs. The reaction mixtures were analyzed by immunoblotting using the antibodies indicated. (**G**) Sperm chromatin was added to mock- or xUsp7-depleted egg extracts. At the time points indicated, chromatin fractions were isolated and subjected to immunoblotting using the antibodies indicated. ∆ Denotes immunodepletion of the protein indicated. Source data are available online for this figure.

Usp7 depletion resulted in the accumulation of ubiquitylated histone H3, and this accumulation was abolished by Usp7/Uhrf1 double depletion (Fig. 4A). Accumulation of ubiquitylated histone H3 was confirmed by the addition of an excess of recombinant His_6_-tagged ubiquitin to egg extracts, showing the slight upward band-shift of ubiquitylated histone H3 (Fig. 4B). A similar accumulation of ubiquitylated histone H3 mediated by Uhrf1 was also observed in Usp7-depleted HeLa cells (Fig. 4C,D). The specificity of the shRNAs used were confirmed by the use of another shRNA for each of targets; this showed similar effects on ubiquitylation of histone H3 (Supplementary Fig. S5). Importantly, both immunopurified xUsp7 from egg extracts and recombinant hUsp7 expressed in insect cells deubiquitylated histone H3 ubiquitylation *in vitro* (Fig. 4E,F). These results suggest that Usp7 functions as a deubiquitylating enzyme toward ubiquitylated histone H3. Consistent with this, Usp7 depletion resulted in the accumulation of Dnmt1 on chromatin (Fig. 4G), as seen when egg extracts were treated with a pan-DUB inhibitor (see Fig. 1C and Supplementary Fig. S3A). Reintroduction of purified recombinant hUsp7 compromised the accumulation of Dnmt1 on chromatin (Supplementary Fig. S6). Although previous work reported that Usp7 stabilizes Dnmt1 by preventing Dnmt1 polyubiquitylaton in mammalian cells^22^, and we also reproduced this observation (Supplementary Fig. S7A), Usp7 depletion did not affect the level and ubiquitylation of Dnmt1 in egg extracts (Supplementary Fig. S7B,C).

**Figure 5:**
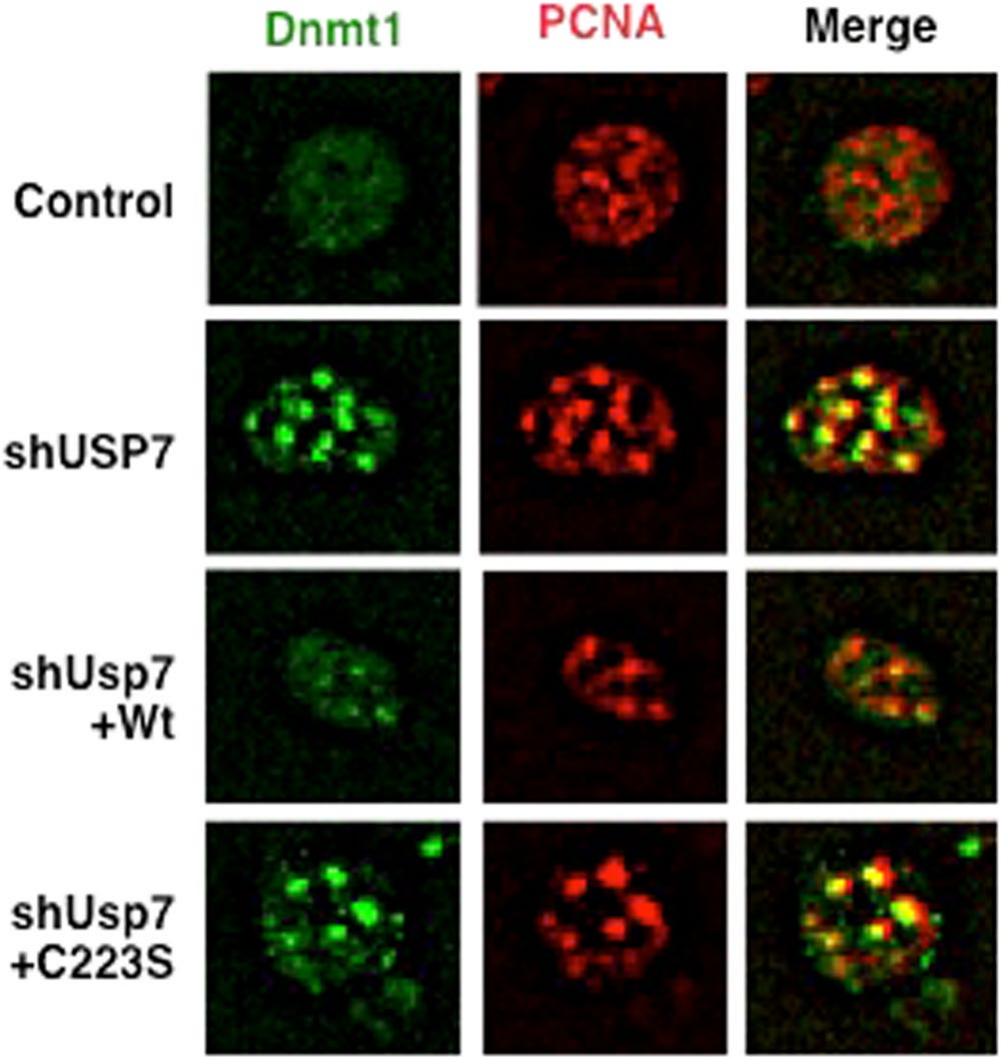
Usp7 is involved in recruitment of Dnmt1 to DNA replication sites in mammals. HeLa cells expressing shRNA-resistant wild-type Usp7(Wt) or its mutant (C223S) were infected with lentiviruses expressing tet-on shRNAs targeting Luciferase (Control) or Usp7 and treated with doxycycline (1 μg/ml) for 72 hrs. The resultant cells were subjected to immunostaining using anti-Dnmt1 (green) and anti-PCNA (red) antibodies.

We further investigated the effect of Usp7 depletion on Dnmt1 recruitment to DNA replication sites in mammalian cells. Dnmt1 nuclear foci co-localized with those of PCNA. Interestingly, Dnmt1 nuclear foci in the absence of Usp7 were much larger and more intense than those in the presence of Usp7. Usp7 depletion did not affect cell cycle progression or DNA replication in mammalian cells (Fig. 5 and Supplementary Fig. S8). Expression of shRNA-resistant wild-type Usp7 in Usp7-depleted cells reduced the size of Dnmt1 nuclear foci, but catalytically-inactive Usp7 (C223S) failed to do so. These results suggest that the DUB activity of Usp7 is likely involved in Dnmt1 recruitment to DNA replication sites (Fig. 5).

### Usp7 regulates maintenance of DNA methylation

**Figure 6:**
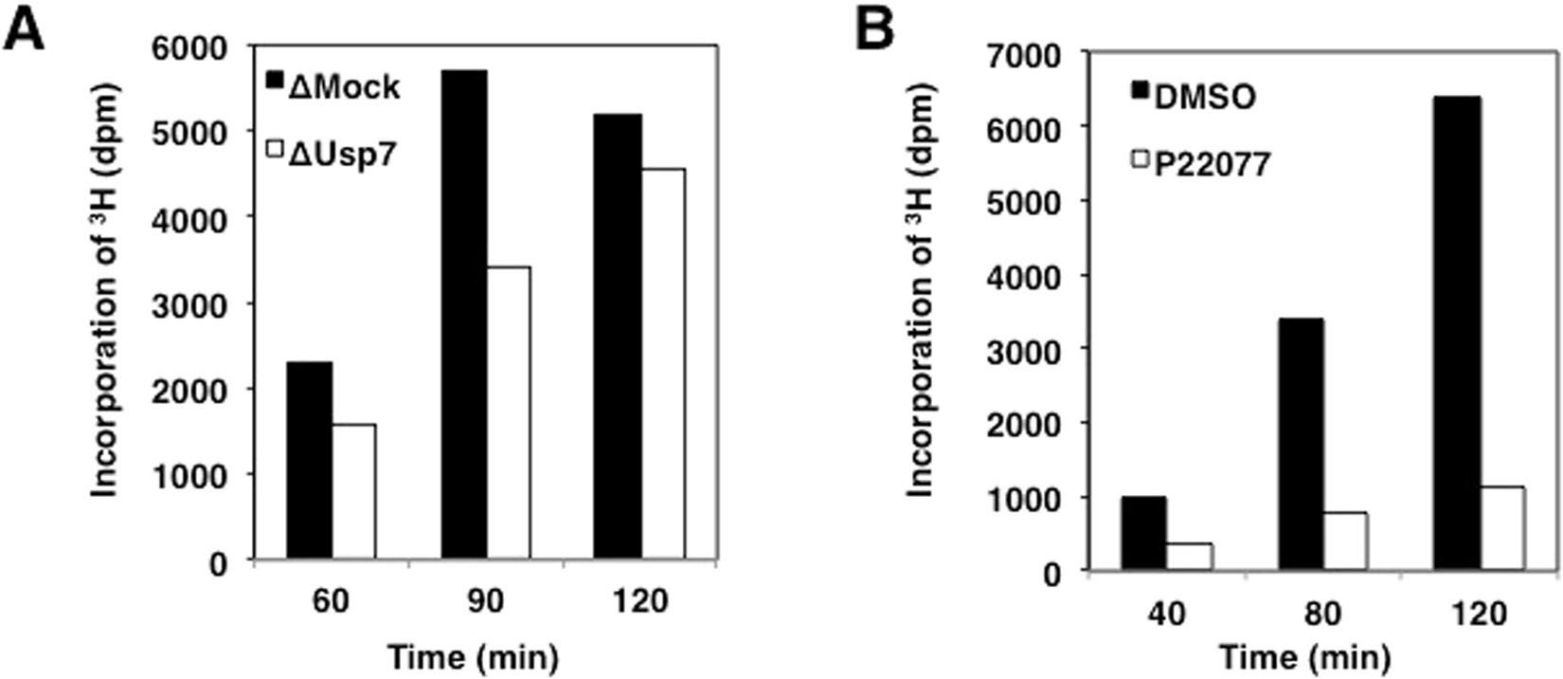
Usp7 plays a key role in efficient DNA methylation. (**A**) Sperm chromatin was added to either mock- or Usp7-depleted extracts in the presence of radiolabelled S-[methyl-^3^H]-adenosyl-L-methionine. The efficiency of DNA methylation was measured at the time points indicated. ∆ Denotes immunodepletion of the protein indicated. (**B**) Interphase egg extracts were treated with 100 μM P22077. The efficiency of DNA methylation was measured at the indicated time points as in (**A**).

We then examined whether Usp7 is involved in the regulation of maintenance DNA methylation. Usp7 depletion caused a delay of DNA-methylation kinetics, without affecting the kinetics and efficiency of DNA replication (Fig. 6A and Supplementary Fig. S9). Consistent with this, treatment with P22077, a specific Usp7 inhibitor, markedly suppressed the level of DNA methylation (Fig. 6B). These observations were also observed in two additional independent experiments (Supplementary Fig. S10A,B). Again, this treatment did not affect DNA replication kinetics and efficiency (Supplementary Fig. S11). Given that a 4A mutant retained a trace ability to bind to Dnmt1, reintroduction of this mutant in endogenous Dnmt1-depleted extracts did not affect DNA methylation (Supplementary Fig. S12). Alternatively, the relatively weak contribution of Usp7 binding of Dnmt1 to DNA methylation maintenance might also be explained by other factors such as Uhrf1 that also recruits Usp7 at DNA replication sites.

## Discussion

**Figure 7:**
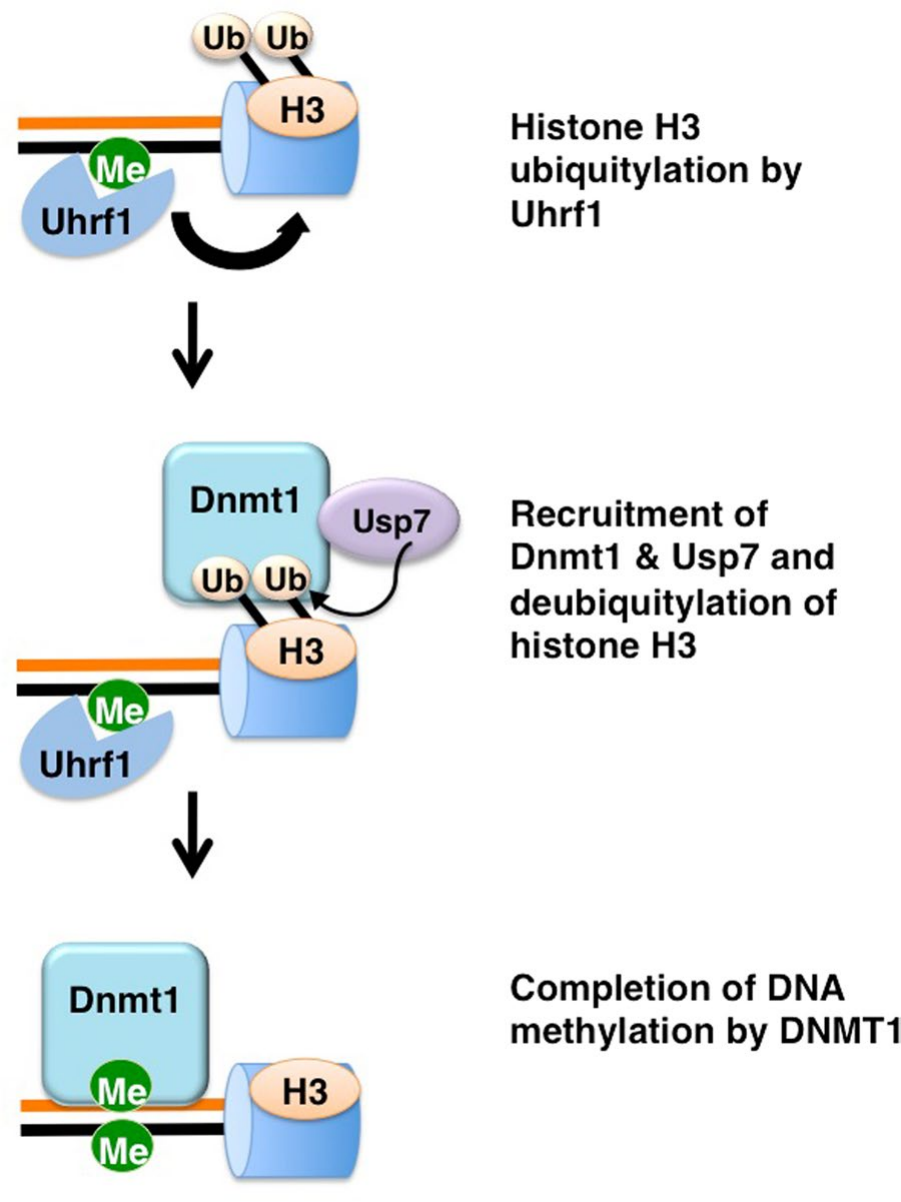
A schematic model showing the role of Usp7 in the maintenance of DNA methylation. During S-phase, Uhrf1 binds to hemi-methylated DNA and ubiquitylates histone H3. Ubiquitylation of histone H3 facilitates the recruitment of Dnmt1/Usp7 complex to DNA methylation sites. Usp7 then deubiquitylates ubiquitylated histone H3, promoting DNA methylation.

In this study, we demonstrate that Usp7 is involved in the regulation of maintenance DNA methylation through deubiquitylation of ubiquitylated histone H3 mediated by Uhrf1 (Fig. 7). This notion is strongly supported by the following observations. 1) Usp7 forms a stable complex with Dnmt1 in both *Xenopus* egg extracts and mammalian cells. 2) Chromatin loading of Usp7 is dependent on ongoing DNA replication and requires both Dnmt1 and Uhrf1 in egg extracts. 3) Usp7 depletion results in marked accumulation of ubiquitylated histone H3 in both egg extracts and mammalian cells. 4) Usp7 depletion in mammalian cells enlarges Dnmt1 nuclear foci during DNA replication. 5) Usp7 inhibition and depletion compromise maintenance DNA methylation in egg extract. It was reported that Usp7 interacts with Dnmt1 and Uhrf1 and stabilize them by preventing their polyubiquitylation and proteasomal degradation^22, 23, 29^. In this respect, we found that inhibition of proteasome activity in *Xenopus* egg extracts did not greatly affect the level of Dnmt1 in egg extracts as well as in their chromatin fractions (Supplementary Figs S3C and S7B). Therefore, polyubiquitylation and subsequent degradation of Dnmt1 are unlikely to affect the accumulation of Dnmt1 on chromatin. Although a recent study also suggested that Usp7-Uhrf1 is also important for the targeting of Uhrf1 to chromatin^30^, our results clearly indicate that chromatin loading of Uhrf1 does not require Usp7, and was rather enhanced by Usp7 depletion, presumably due to suppression of histone H3 deubiquitylation and subsequent conversion from hemi-methylated DNA to fully-methylated DNA.

Consistent with our observations, it has been reported that downregulation of Usp7 in mammalian cells results in a reduction in the level of DNA methylation^22, 24^. Given that Usp7 depletion caused accumulation of ubiquitylated histone H3 and Dnmt1 bound to chromatin, it is likely that Dnmt1-bound Usp7 preferentially targets ubiquitylated histone H3 to release Dnmt1 from ubiquitylated histone H3. Recent structural analyses of Dnmt1 revealed that the RFTS domain of Dnmt1, which is required for interaction with ubiquitylated histone H3, inserted into the catalytic pocket of Dnmt1, leading to autoinhibition of its activity in *cis* ^31, 32^. Given that Dnmt1 association with ubiquitylated histone H3 likely causes a conformational change in the catalytic pocket of Dnmt1, leading to its enzymatic activation, removal of ubiquitin from histone H3 by Usp7 might regulate Dnmt1’s catalytic activity. Another possibility is that the tight interaction between Dnmt1 and ubiquitylated histone H3 hinders processive DNA methylation during DNA replication^12, 13^.

Although Usp7 is a key factor for deubiquitylation of ubiquitylated histone H3, when compared with the treatment with P22077, the effect of Usp7 depletion on suppression of histone H3 deubiquitylation appeared incomplete, ultimately showing a loss of ubiquitylated histone H3, chromatin-bound Dnmt1 and Uhrf1. The inhibition of DNA methylation by Usp7 depletion was also only partial. These results suggest that other unidentified P22077-sensitive DUB(s) are also involved in the regulation of the level of histone H3 ubiquitylation and DNA methylation. In this regard, P22077 was reported to inhibit Usp47. Therefore we examined the possibility that Usp47 is also involved in Dnmt1 recruitment to DNA replication sites and subsequently DNA methylation maintenance. Immunocytochemical analyses revealed that depletion of Usp47 did not affect Dnmt1 focus formation, whereas depletion of Usp7 enlarged them (Supplementary Fig. S13A,B). The double depletion did not further affect the enlargement of Dnmt1 nuclear foci in Usp7-depleted cells. Taken together, the results suggest that Usp47 is not a major deubiquitylating enzyme that regulates recruitment of Dnmt1 to DNA replication sites. However, considering the fact that both ubiquitylated H2A and H2B are targeted by multiple DUBs^20, 21^, it would be of great interest to identify other DUBs that target ubiquitylated histone H3 and regulate DNA methylation.

## Methods

### *Xenopus* egg extracts, DNA replication, and methylation assays

Preparation of cytostatic factor (CSF)-arrested extracts, interphase egg extracts (low-speed supernatants [LSS] and high-speed supernatants [HSS]), chromatin isolations, replication assays, DNA methylation assays, and immunodepletions were performed as described previously^16^. All extracts were supplemented with energy regeneration mix (2 mM ATP, 20 mM phosphocreatine, and 5 μg/ml creatine kinase). Demembranated sperm nuclei (3,000–4,000 sperm/μl in the final reaction) were added to egg extracts and incubated at 22 °C. Each replication or methylation assay was repeated at least three times, and representative results are shown. For chromatin spin-downs from LSS, sperm nuclei were incubated in 15–40 μl of extracts. Extracts were diluted with 200 μl of ice-cold 2% CPB (50 mM KCl, 2.5 mM MgCl_2_, 20 mM HEPES-KOH, pH 7.7) containing 2% sucrose, 0.1% NP-40, and kept on ice for 5 min. Diluted extracts were layered over 1.5 ml of 30% sucrose cushion in CPB and centrifuged at 15,000 × g for 10 min at 4 °C using a swing–bucket rotor. The pellets were resuspended in Laemmli sample buffer. For Ub-VS reactions, LSS was incubated with 20 μM Ub-VS (Boston Biochem, Cambridge, MA, USA) for 30 min at 22 °C. Then sperm nuclei were added to egg extracts with or without 58 μM ubiquitin (Boston Biochem). PR-619 (100 μM, Sigma-Aldrich) was also used to inhibit DUB activities in egg extracts. For other inhibitor studies the following reagents were added as indicated: To inhibit DNA replication, LSS was pre-incubated with geminin (400 nM), p27^Kip1^ (5 μM), or Aphidicolin (150 μM) for 10 min. When indicated, LSS was pre-incubated with P22077 (100 μM) prior to sperm nuclei addition. MG132 (100 μM) was used to inhibit the proteasome.

### Immunological methods

Antibodies against Usp7, Dnmt1, Uhrf1, PCNA, histone H3, Cdt1, Ubiquitin, and FLAG were used as described previously^16, 33^. Dnmt1- or Uhrf1-depletion of LSS and HSS were carried out as previously described. To deplete Usp7 from *Xenopus* egg extracts, 30 μg of anti-Usp7 antibodies or control IgG was coupled with 40 μl rProtein A Sepharose (GE Healthcare) overnight at 4 °C. The beads were washed three times with 400 μl CPB containing 2% sucrose. 1 volume of LSS was incubated with 0.2 volumes of antibody beads and incubated for 1 hr at 4 °C. The depletion procedure was repeated once. For immunoprecipitation, 10 μl of Protein A agarose (GE Healthcare) was coupled with 2 μg of purified antibodies or control IgG. The agarose beads were washed twice with CPB containing 2% sucrose. The antibody beads were incubated with HSS and incubated for 1 hr at 4 °C. The beads were washed four times with IP buffer (20 mM HEPES-KOH, pH 7.7, 50 mM KCl, 2.5 mM MgCl_2_, 0.1% Triton X-100) and resuspended in 20 μl of 2 × Laemmli sample buffer. Immunoprecipitation of chromatin-bound histone H3 was performed as described previously^33^ (see Supplementary Method). In experiments examining the interaction between recombinant xDnmt1 and Usp7, HSS was supplemented with 85 nM recombinant xDnmt1 (wild-type or KG linker mutant, see below) or reticulocyte lysate expressing xDnmt1 fragments and subjected to immunopreciptation using anti-Usp7 or FLAG antibodies.

To identify Dnmt1-interacting proteins, the Dnmt1 immunoprecipitates were separated in a 4–12% gradient SDS-polyacrylamide gel and then each lane was cut into nine pieces for liquid chromatography coupled to tandem MS (LC-MS/MS). Data analysis for LC-MS/MS was performed as described previously^34, 35^, with minor modifications. The LC-MS/MS data were analyzed using Mascot (Matrix Science) and searched against a *Xenopus laevis* subset database created from UniProt (release-2013_01). The resulting files were loaded into Scaffold software (Proteome Software Inc.) for comparing identified proteins between samples.

### Cloning of *Xenopus* Usp7

A cDNA library from *Xenopus laevis* eggs was prepared using SMARTer RACE cDNA Amplification Kit (Clontech) and used for PCR amplification of the Usp7 cDNA with the primers 5′-CCCAGTCTGGAAACATGAACCAC-3′ and 5′-CTCATATTTTAGATTTTGGCACACAC-3′. The sequence of *Xenopus* Usp7 cDNA was deposited to the GenBank database (accession number KX518317).

### Recombinant Protein expression and purification

For protein expression in a rabbit reticulocyte lysates (RRL) system, the xDnmt1 genes were transferred into pKS104 vector as described below. To generate xDnmt1 fragments corresponding to amino acid residues 1–1490, 180–1490, 471–1490, 1–470, 1–981, 585–1490, 780–1490 and 981–1490, PCR was performed using the following sets of primers: 5′-TTGCACTCGAGAATTCATGGCGGCCCAGTCCACT-3′ and 5′-TTTGTAGTCGGTCGACTCATCTGTTTCCATCTTTTCA-3′, 5′-TTGCACTCGAGAATTCATGGATGCAGACCAACCAG-3′ and 5′-TTTGTAGTCGGTCGACTCATCTGTTTCCATCTTTTCA-3′, 5′-TTGCACTCGAGAATTCATGCGAGCAGCAAGAAGAC-3′ and 5′- TTTGTAGTCGGTCGACTCATCTGTTTCCATCTTTTCA-3′, 5′-TTGCACTCGAGAATTCATGGCGGCCCAGTCCACT-3′ and 5′-TTTGTAGTCGGTCGACCTTTTGCCCAGAGTAACAC-3′, 5′-TTGCACTCGAGAATTCATGGCGGCCCAGTCCACT-3′ and 5′-TTGCACTCGAGAATTCATGGTTAACAAAGGCAAAGGAAAG-3′, 5′-TTGCACTCGAGAATTCATGCCATCACCCAAAAAGAT-3′ and 5′-TTTGTAGTCGGTCGACTCATCTGTTTCCATCTTTTCA-3′, 5′-TTGCACTCGAGAATTCATGATACCCAGAGTAAGCAATCC-3′ and 5′-TTTGTAGTCGGTCGACTCATCTGTTTCCATCTTTTCA-3′, respectively. The resulting DNA fragment was cloned into pKS104 vector using the In-Fusion Cloning Kit (Clontech), generating the constructs pKS104-xDnmt1(1–1490), pKS104-xDnmt1(180–1490), pKS104-xDnmt1(471–1490), pKS104-xDnmt1(1–470), pKS104-xDnmt1(1–981), pKS104-xDnmt1(1–1490), pKS104-xDnmt1(585–1490), pKS104-xDnmt1(780–1490), pKS104-xDnmt1(981–1490). Proteins were expressed using TNT SP6 Quick Master Mix (Promega) following the manufacturer’s protocol.

For protein expression in insect cells, xDnmt1 was cloned into the pKS104 vector as described previously, and transferred into pVL1392 using primers 5′-GGCGCGGATCAGATCTGATGGCGGCCCAGTCCACT-3′, and 5′-GGGCCCTCTAGAATTCTACTTGTTATCGTCATCCT-3′. An xDnmt1 KG linker (4A) mutant was generated using the KOD-Plus Mutagenesis Kit (Toyobo) with primers 5′-GCCTTTGTTAACAGCACCGCGAGC-3′, and 5′-GCAGGAGCGGGCGCAGGAGCAGGCAAAGGCAAAACTCCTTCAAAG-3′. Baculoviruses were produced using the BD BaculoGold Transfection Kit (BD Biosciences), following the manufacturer’s protocol. Proteins were expressed in Sf9 insect cells by infection with viruses expressing xDnmt1 Wt-FLAGx3 or xDnmt1 KG linker mutant-FLAGx3 for 72 hrs. Sf9 cells from 500 ml culture were collected and lysed by resuspending in 20 ml lysis buffer (20 mM Tris-HCl, pH 8.0, 100 mM KCl, 5 mM MgCl_2_, 10% glycerol, 1% NP-40, 1 mM DTT, 10 μg/ml leupeptin, and 10 μg/ml aprotinin), followed by incubation on ice for 10 min. A soluble fraction was obtained after centrifugation of the lysate at 15,000 × *g* for 15 min at 4 °C. The soluble fraction was incubated for 4 hrs at 4 °C with 250 μl of anti-FLAG M2 affinity resin (Sigma-Aldrich) equilibrated with lysis buffer. The beads were collected and washed with 10 ml wash buffer (20 mM Tris-HCl, pH 8.0, 100 mM KCl, 5 mM MgCl_2_, 10% glycerol, 0.1% NP-40, 1 mM DTT) and then with 5 ml EB (20 mM HEPES-KOH, pH 7.5, 100 mM KCl, 5 mM MgCl_2_) containing 1 mM DTT. The recombinant xDnmt1 was eluted in 250 μl EB containing 1 mM DTT and 250 μg/ml 3 × FLAG peptide (Sigma-Aldrich) twice. Eluates were pooled and concentrated.

### Cell culture, virus generation and infection

HeLa cells were cultured in DMEM (Gibco/Thermo Fisher Scientific) supplemented with 10% fetal bovine serum. Lentiviruses expressing the respective shRNAs or genes were generated by co-transfection of 293T cells with pCMV-VSV-G-RSV- RevB, pCAG-HIVgp and the respective CS-RfA-ETBsd, CS-RfA-ETHygro, CS-RfA-ETPuro, CS-IV-TRE-RfA-UbC-Puro or CS-IV-TRE-RfA-UbC-Hygro using the calcium phosphate co-precipitation method. Cells infected with the viruses indicated were treated with 10 μg/ml of blasticidin (Gibco/Thermo Fisher Scientific), and/or 2 μg/ml of puromycin (Sigma-Aldrich) for 2–3 days. Doxycycline (Sigma-Aldrich) was added to the medium at a concentration of 1 μg/ml for inducible expression of the respective shRNAs or genes. To generate wild-type or C223S Usp7-expressing cells, plasmids that are shRNA resistant (see below) were transfected into Usp7 knockdown HeLa cells using Lipofectamine 2000 (Invitrogen/Thermo Fisher Scientific).

### Plasmid construction

To generate lentivirus-based shRNA constructs, a 19–21 bp shRNA-coding fragment containing a 5′-ACGTGTGCTGTCCGT-3′ loop was introduced into pENTR4-H1 digested with *Age*I/*Eco*RI. To insert the H1tetOx1-shRNA into a lentivirus vector, we mixed the resulting pENTR4-H1- shRNA vector and CS-RfA-ETBsd vector with Gateway LR clonase (Invitrogen/Thermo Fisher Scientific). All the target sequences for lentivirus-based shRNAs are described in Supplementary Table S1. To construct the plasmid for expression of shRNA-resistant hUsp7, full-length hUsp7 cDNA was inserted into pCI-neo vector with FLAG-epitope and mutagenized using the KOD-Plus Mutagenesis Kit, with the following primers: 5′-ATATACCGTATTTAAGGTATTGAAGaactcctcgcttgctg-3′ and 5′-ACATGTACGATGAAGAAAAAGTGAA-3′. The C223S mutation was introduced using the primers 5′-AGTTACATGAACAGCCTGCTACAG-3′ and 5′-AGTCGCTCCCTGATTCTTTAAGCCG-3′.

### Immunofluorescence analysis

For the immunofluorescence assay for Dnmt1 and PCNA in HeLa cells, the cells were harvested 48 hrs after transfection. Cells were treated with CSK buffer (100 mM NaCl, 300 mM sucrose, 10 mM PIPES (pH 6.8), 3 mM MgCl_2_, 1 mM EGTA) containing 0.5% Triton X-100 for 3 min, fixed with 4% paraformaldehyde for 10 min and then treated with methanol for 20 min at −20 °C. The cells were blocked with 5% BSA for 30 min at RT, followed by incubation with antibodies against Dnmt1 and PCNA at 1:100 dilution for 2 hrs at RT, and incubated with secondary antibodies. Lastly, cells were treated with Hoechst 33342 (1 μg/ml) for 10 min.

### Cell cycle analysis

For DNA content analysis, control or Usp7-depleted HeLa cells were fixed by incubation in 70% ethanol overnight. Cells were washed with PBS and treated with PBS containing RNase A (0.1 mg/ml) and propidium iodide (PI) (50 μg/ml) for 30 min at 37 °C, then analyzed by flow cytometry.

All other experimental details are described in the Supplementary Information.

## Electronic supplementary material

Supplementary Figure S1-S13, Supplementary Table S1, Supplementary Method
