# Supplementary material for: Usp7-dependent histone H3 deubiquitylation regulates maintenance of DNA methylation
Source: Sci Rep. 2017 Mar 3;7:55. doi: 10.1038/s41598-017-00136-5 (PMC5427934; doi:10.1038/s41598-017-00136-5)
Supplement: Supplementary file 1 — Supplementary Figure S1-S13, Supplementary Table S1, Supplementary Method [file 41598_2017_136_MOESM1_ESM.pdf]

Usp7-dependent histone H3 deubiquitylation regulates maintenance of DNA methylation

Luna Yamaguchi, Atsuya Nishiyama**, Toshinori Misaki, Yoshikazu Johmura, Jun Ueda, Kyohei Arita, Koji Nagao, Chikashi Obuse, Makoto Nakanishi*

*E-mail: mkt-naka@ims.u-tokyo.ac.jp
**E-mail: anishiya@ims.u-tokyo.ac.jp

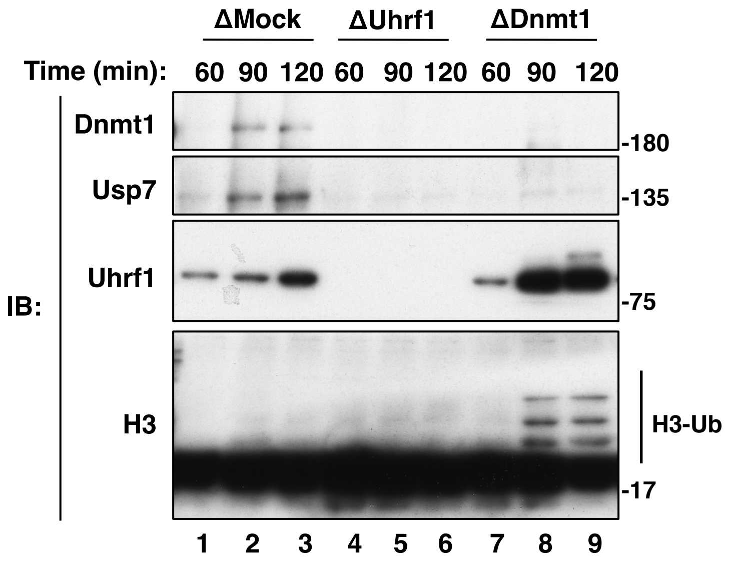

Supplementary Figure S1. Accumulation of ubiquitylated H3 in Dnmt1-depleted chromatin

Sperm chromatin was added to mock-, Uhrf1-, or Dnmt1-depleted interphase extracts. At the indicated time points, chromatin fractions were isolated and subjected to immunoblotting using the antibodies indicated.

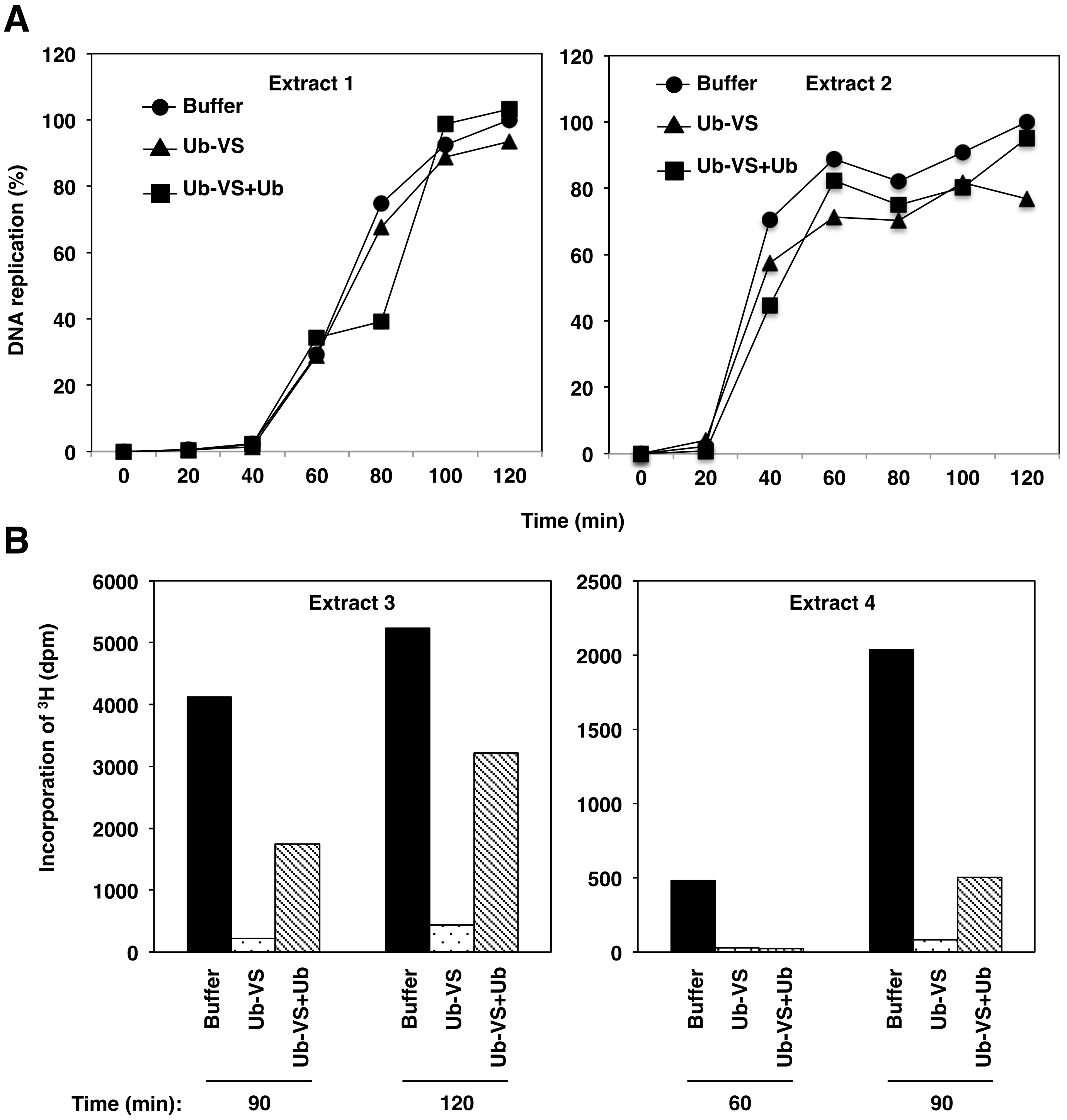

Supplementary Figure S2. DUB activity is important for maintenance of DNA methylation, but not for DNA replication, in *Xenopus* egg extracts

Sperm chromatin was incubated with interphase *Xenopus* egg four independent extracts (Extracts 1 to 4)supplemented with buffer (+buffer), 20 μM Ub-VS (+Ub-VS), or 20 μM Ub-VS and 0.2 mg/ml ubiquitin (+Ub-VS+Ub). Prior to addition of sperm chromatin, radiolabeled [α-^{32}P]dCTP (A) or S-[methyl-^{3}H]-adenosyl-L-methionine (B) was added to extracts. Purified DNA samples were analyzed to determine the efficiency of DNA replication (A) and DNA methylation (B), as described in Experimental Procedures.

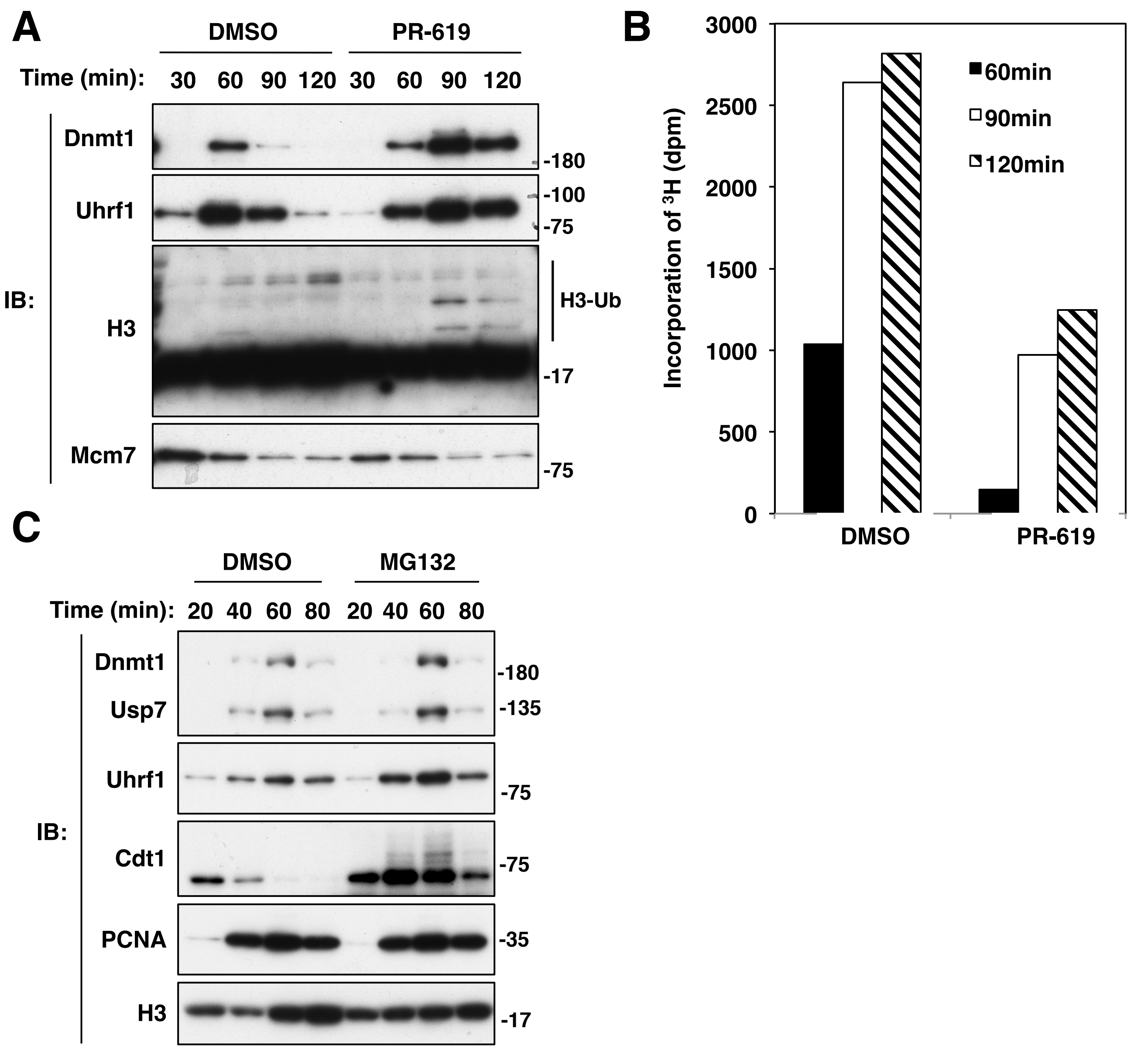

Supplementary Figure S3. PR-619, a general DUB inhibitor, suppresses maintenance DNA methylation

(A) Sperm chromatin was added to control (DMSO) or PR-619-treated extracts. At the time points indicated, chromatin fractions were isolated and subjected to immunoblotting using the antibodies indicated. (B) DNA methylation assay using *Xenopus* egg extracts in the presence of PR-619 (100 μM). Sperm chromatin and S-[methyl-^{3}H]-adenosyl-L-methionine were added to PR619-treated extracts. DNA methylation was evaluated by incorporation of ^{3}H in the sperm DNA at the indicated times. (C) Inhibition of proteasome activity suppresses Cdt1 degradation, but does not affect DNA methylation. Sperm chromatin was added to control (DMSO) or MG132 (100 μM)-treated extracts 15 min after treatment. Chromatin fractions were isolated at the indicated times and subjected to immunoblotting using the antibodies indicated. In these extracts, the half-life of the Dnmt1 protein appeared to be more than 2 hrs. The half-life of Cdt1 was also more than 2 hrs under non-DNA replication conditions, but was about 0.5-1 hr under DNA replication conditions.

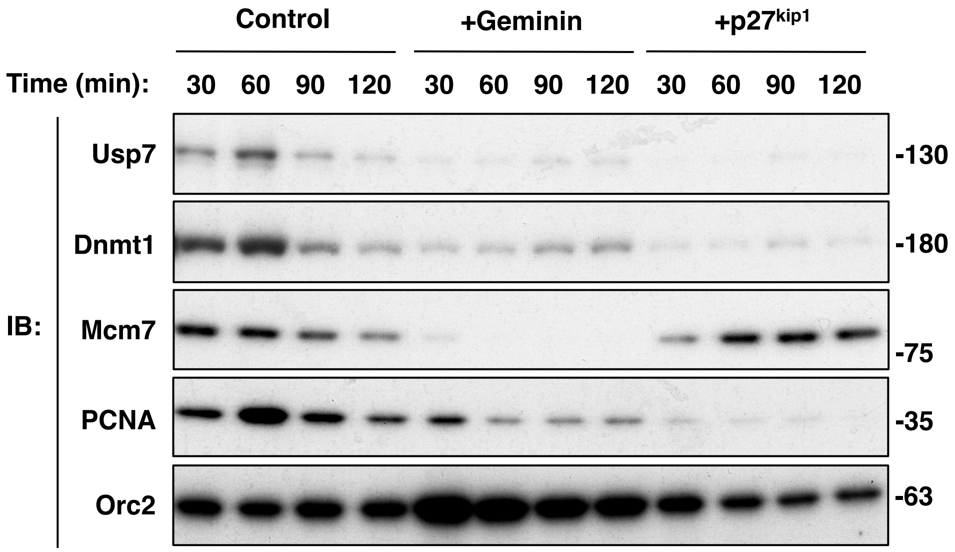

Supplementary Figure S4. USP7 chromatin binding requires DNA replication

Sperm chromatin was added to egg extracts in the presence or absence of Geminin or p27^{kip1}. Chromatin fractions were isolated at the indicated time points and subjected to immunoblotting using the antibodies indicated. Control (untreated) extracts were also analyzed.

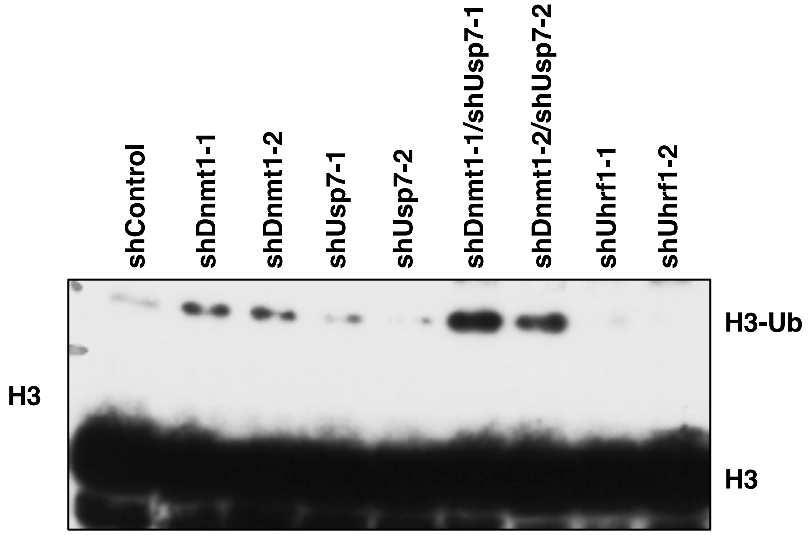

Supplementary Figure S5. Effect of different Dnmt1, Usp7 and Uhrf1 shRNAs on histone H3 ubiquitylation

HeLa cells were infected with lentiviruses expressing Tet-On shRNAs targeting Luciferase (Control), Dnmt1, Usp7, and Uhrf1, in the combinations indicated. Cells were then harvested after treatment with doxycycline (1 μg/ml) for 3 days. Histones were acid-extracted from cells depleted of the indicated proteins and subjected to immunobloting using anti-histone H3 antibodies.

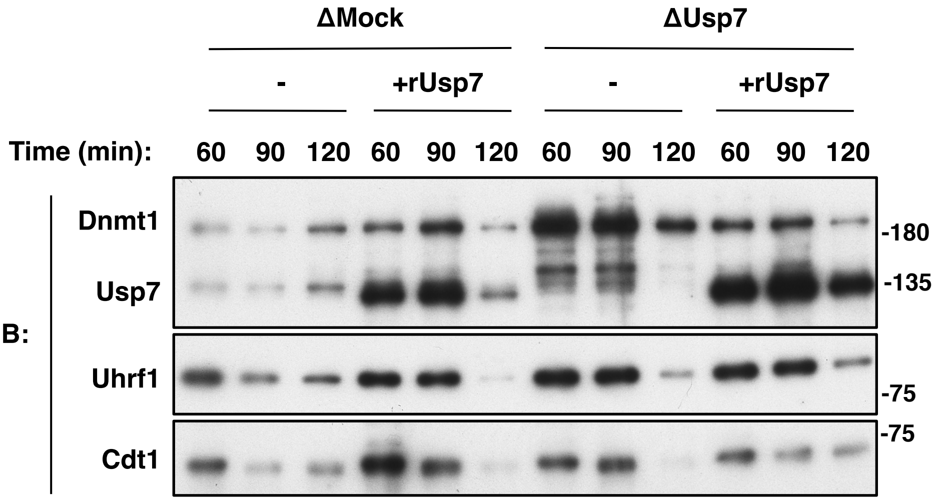

Supplementary Figure S6. Recombinant Usp7 restores chromatin loading of Dnmt1 in Usp7-depleted extracts

Sperm chromatin was incubated with mock (ΔMock)- or USP7-depleted extracts in the presence or absence of purified recombinant Usp7. At the indicated time points, isolated chromatin fractions were subjected to immunoblotting using the antibodies indicated.

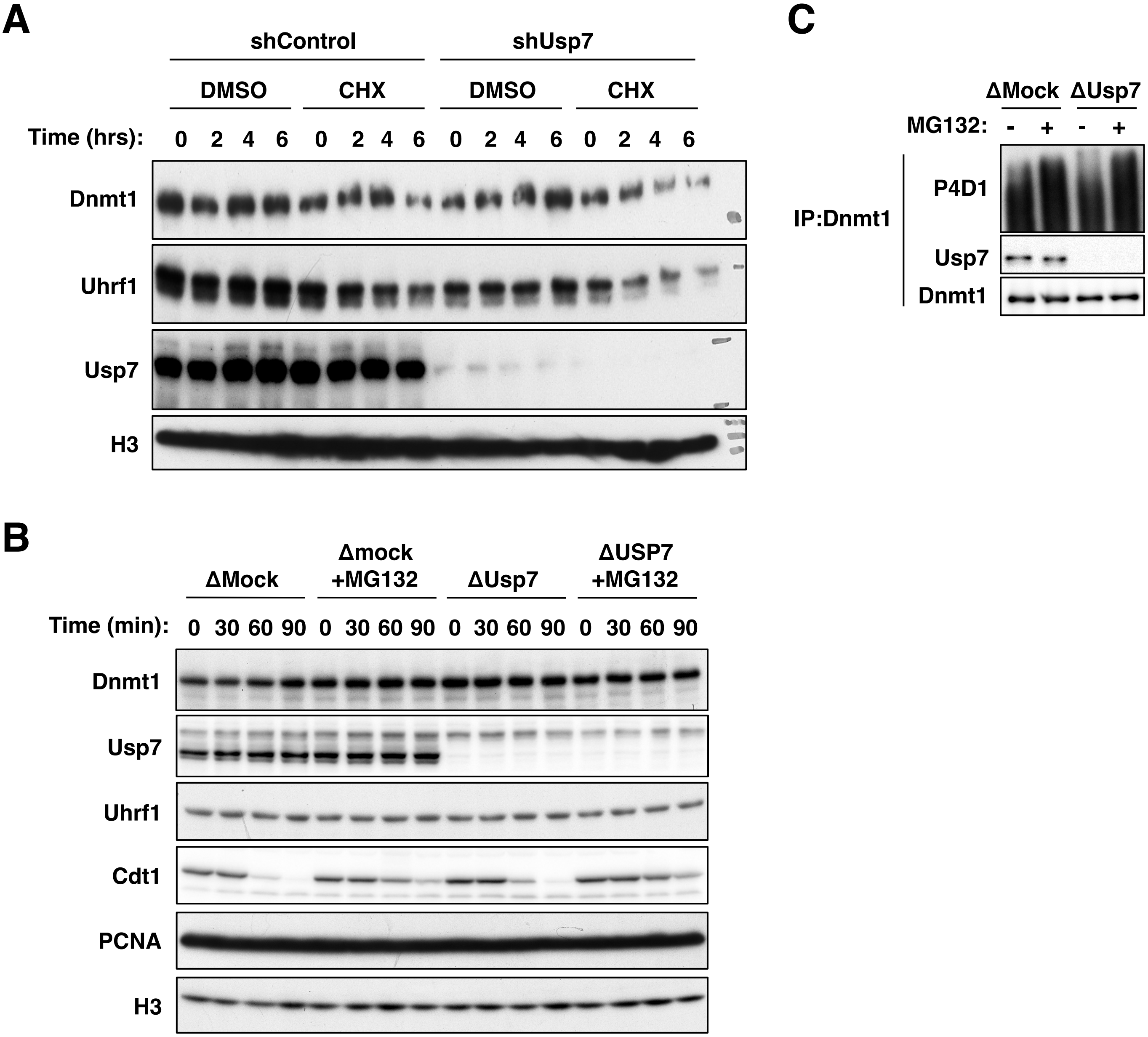

Supplementary Figure S7. Usp7 depletion did not affect the level of Dnmt1 and its ubiquitylation in egg extracts, but regulated the level of Dnmt1 in mammalian cells

(A) HeLa cells were infected with expressing Tet-On shRNAs targeting Luciferase (Control), or Usp7. After treatment with doxycycline (1 μg/ml) for 3 days, cells were treated with cycloheximide (100 μg/ml) and harvested at different time points. Whole cell extracts were subjected to immunoblotting using the antibodies indicated. (B) Sperm chromatin was added to either mock- or Usp7-depleted extracts in the presence or absence of 100 μM MG132. Total egg extracts were isolated at indicated time points and subjected to immunoblotting using the antibodies indicated. (C) Mock- or Usp7-depleted extracts were incubated at 22˚C for 90 min in the presence or absence of 100 μM MG132 and subjected to immunoprecipitation using the xDnmt1 antibodies. Immunoprecipitated proteins were analyzed by immunoblotting using the antibodies indicated.

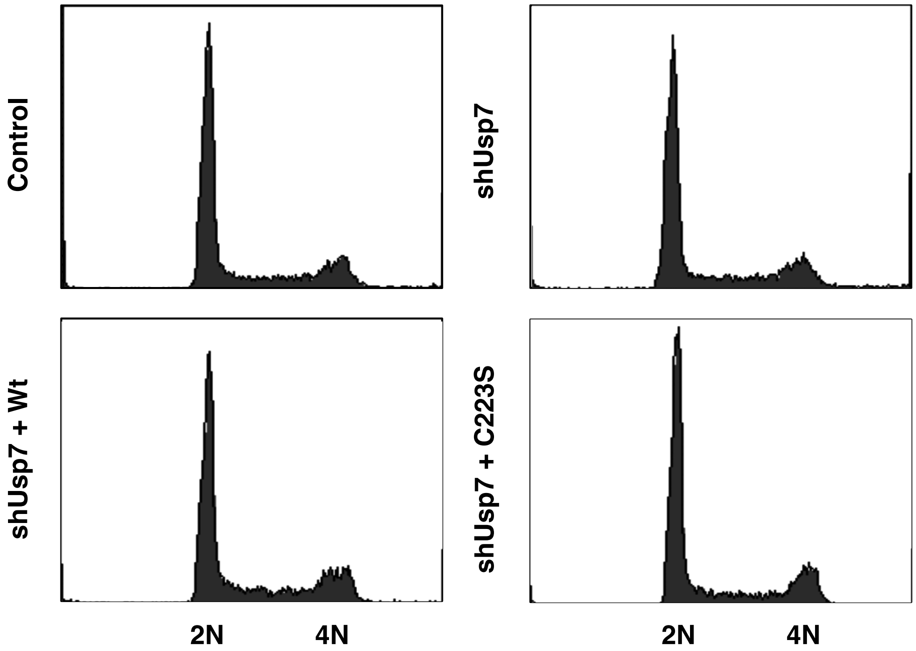

Supplementary Figure S8. Usp7 depletion does not affect cell cycle progression

HeLa cells expressing either wild-type (Wt) or a mutant (C223S) hUsp7 were infected with lentiviruses expressing Control (Luciferase) or Usp7 shRNA. Cells were cultured in the presence of doxycyclin (1 μg/ml) and stained with propidium iodide (PI). The cell cycle profile was analyzed by flow cytometry.

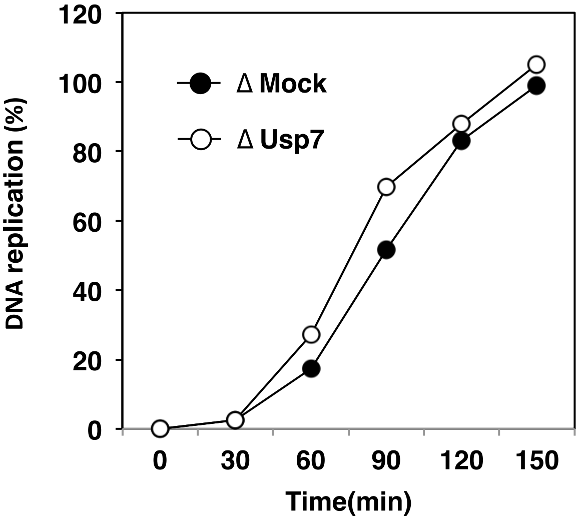

Supplementary Figure S9. Usp7 depletion from *Xenopus* egg extracts does not inhibit DNA replication

Sperm chromatin was added to mock- or Usp7-depleted extracts in the presence of radiolabled dCTP. The resultant extracts were subjected to DNA replication assay at the time points indicated.

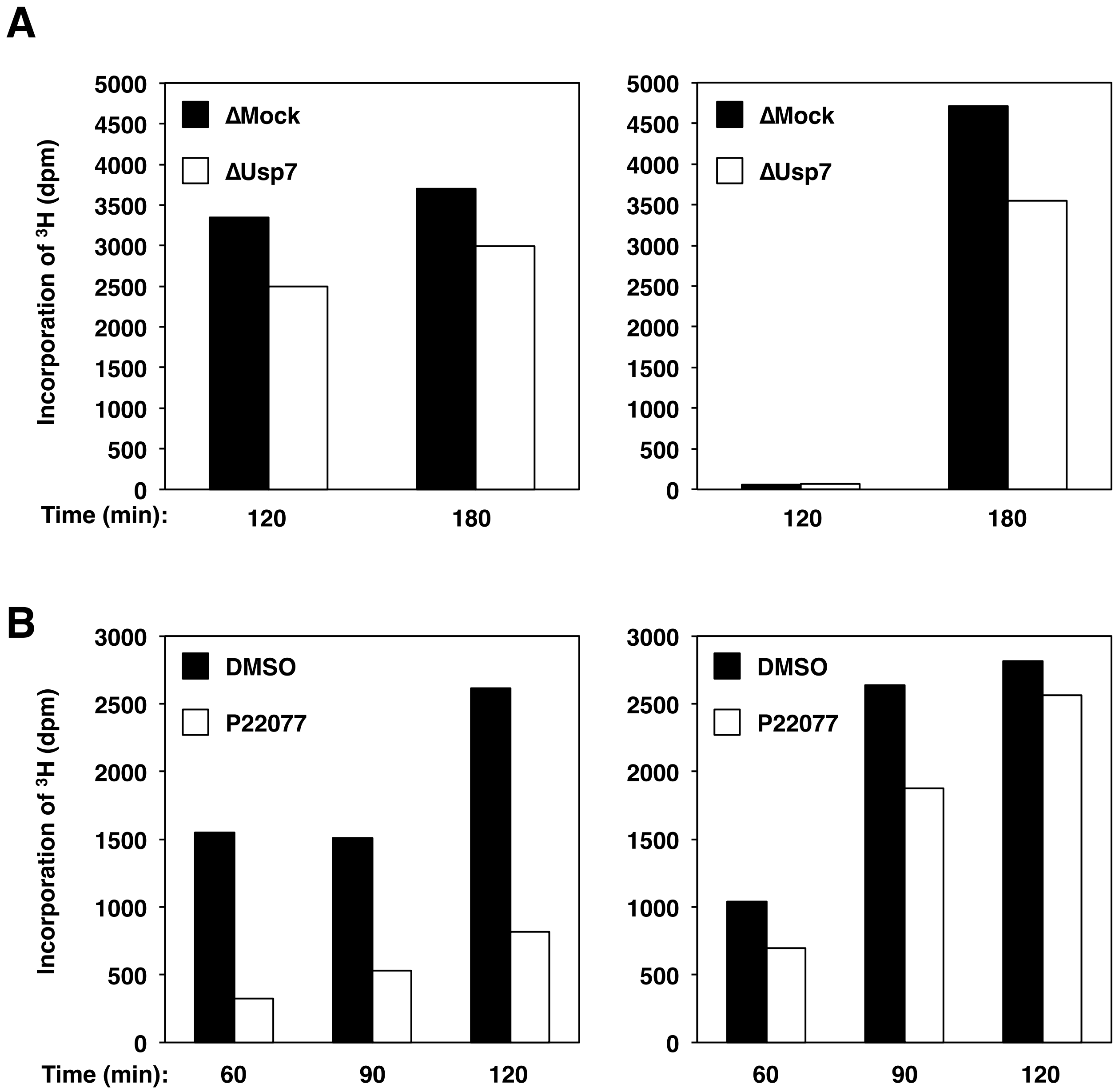

Supplementary Figure S10. Usp7 plays a key role in efficient DNA methylation

(A) Sperm chromatin was added to either mock- or Usp7-depleted extracts in the presence of radiolabelled S-[methyl-^{3}H]-adenosyl-L-methionine. The efficiency of DNA methylation was measured at the time points indicated. (B) Interphase egg extracts were treated with 100 μM P22077. The efficiency of DNA methylation was measured at the indicated time points as in (A and B).

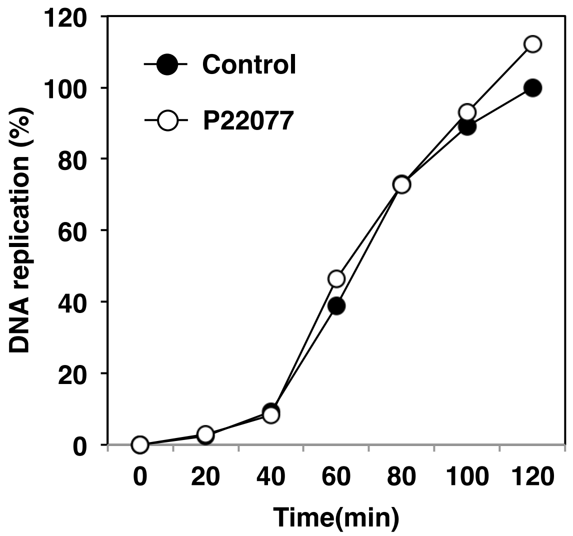

Supplementary Figure S11. P22077 treatment of *Xenopus* egg extracts does not inhibit DNA replication

Sperm chromatin was added to interphase egg extracts with DMSO (Control) or P22077 in the presence of radiolabled dCTP. The resultant extracts were subjected to DNA replication assay at the time points indicated.

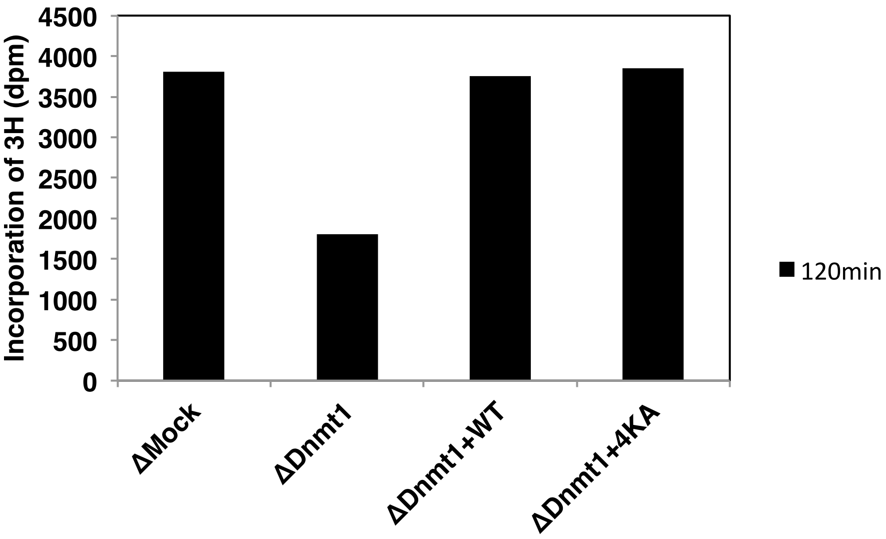

Supplementary Figure S12. Dnmt1-4KA mutant supports DNA methylation

Sperm chromatin was added to interphase extracts that were mock depleted or immunodepleted of DNMT1. xDNMT1-depleted extracts were supplemented with either buffer alone, purified recombinant xDNMT1 WT or 4KA mutant-FLAGx3 (10 ng/µl final concentration, respectively). At the indicated time point, Purified DNA samples were analyzed to determine the efficiency of DNA methylation

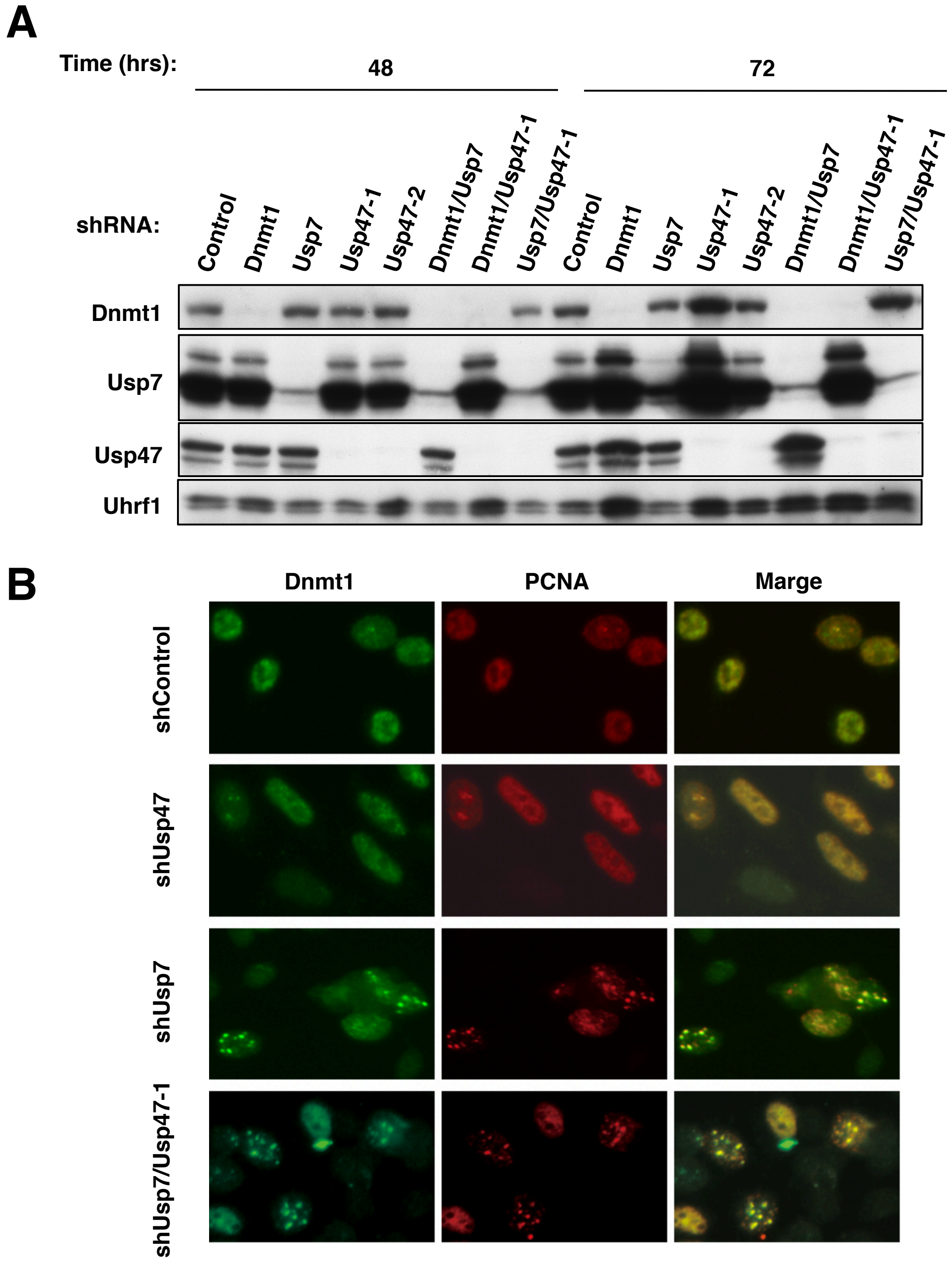

Supplementary Figure S13. Knock-down of Usp47 in HeLa cells does not affect Dnmt1recruitment to DNA replication sites

(A) HeLa cells were infected with lentiviruses expressing Tet-On shRNAs targeting Luciferase (Control), Dnmt1, Usp7, and Usp47, in the combinations indicated. Cells were then harvested after treatment with doxycycline (1 μg/ml) for 2 or 3 days, and whole cell extracts were subjected to immunoblotting using the antibodies indicated. (B) The resultant cells were subjected to immunostaining using anti-Dnmt1 (green) and anti-PCNA (red) antibodies.

shRNA sequences used in this study

Target gene	Sequence	Reference	Type
DNMT1	GGAGAACGGTGCTCATGCTT	Unterberger et al., 2006	Lentivirus
DNMT1	CGAGTTGCTAGACCGCTTC	Suzuki eta al., 2004	Lentivirus
USP7	GGAGTTTGAGAAGTTTAAA	Kessler et al., 2007	Lentivirus
USP7	ATACACTGTGTTCAAAGTA	Kessler et al., 2007	Lentivirus
UHRF1	GCGCTGGCTCTCAACTGCTTT	Rajakumara et al., 2011	Lentivirus
UHRF1	GCCTTTGATTCGTTCCTTCTT	Scott et al., 2012	Lentivirus

Supplementary Table S1

Supplementary Method

Immunoprecipitation of chromatin-bound histone H3

For immunodepletion of xDnmt1 from *Xenopus* egg extracts, 250 μl of antiserum against xDnmt1 or preimmune serum were conjugated to 40 μl of recombinant protein A Sepharose beads (GE Healthcare). Then, 20 μl of beads were incubated with 100 μl LSS and incubated at 4˚C for 1 hr. This procedure was repeated twice. Mock- or xDnmt1-depleted extracts were incubated with sperm chromatin at 22˚C for 90-150 min. After incubation, chromatin fractions were isolated as described previously[1,2]. The chromatin pellet was then resuspended and digested in 100 μl digestion buffer (10 mM HEPES-KOH, 50 mM KCl, 2.5 mM $MgCl_2$, 0.1 mM $CaCl_2$, 0.1% Triton X-100, pH 7.5) containing 4 U/ml micrococcal nuclease (MNase) at 22˚C for 20 min. The reaction was stopped by the addition of 10 mM EDTA, and the mixture was centrifuged at 15,000 rpm for 10 min. For H3 immunoprecipitation, the supernatant was treated with 1% SDS to denature histones, and then immediately diluted with lysis buffer (150 mM NaCl, 1% Triton X-100, 1 mM EDTA, 15 mM Tris-HCl, pH 8.0). Diluted supernatants were subjected to immunoprecipitation using anti-H3 antibody conjugated to Protein A agarose (Roche). For the pull-down experiment, immunoprecipitated histone H3 was incubated with mock or Dnmt1-depleted HSS for 1 hr at

4°C. Beads were washed four times with CPB containing 0.1% Triton X-100, and bound proteins were eluted with Laemmli sample buffer and analyzed by SDS-PAGE.

Supplementary References

1. Nishiyama, A. *et al.* Uhrf1-dependent H3K23 ubiquitylation couples maintenance DNA methylation and replication. *Nature* 502, 249–253 (2013).
2. Misaki, T. *et al.* The replication foci targeting sequence (RFTS) of DNMT1 functions as a potent histone H3 binding domain regulated by autoinhibition. *Biochem. Biophys. Res. Commun.* 470, 741–747 (2016).
